# Dynamic changes in the physicochemical properties of fresh-cut produce wash water as impacted by commodity type and processing conditions

**DOI:** 10.1371/journal.pone.0222174

**Published:** 2019-09-26

**Authors:** Jie Li, Zi Teng, ShihChi Weng, Bin Zhou, Ellen R. Turner, Bryan T. Vinyard, Yaguang Luo

**Affiliations:** 1 College of Food Science and Technology, Huazhong Agricultural University, Wuhan, PR China; 2 U. S. Department of Agriculture, Agricultural Research Service, Beltsville Agricultural, Research Center, Environmental Microbiology and Food Safety Laboratory, Beltsville, MD, United States of America; 3 Department of Nutrition and Food Science, University of Maryland, College Park, MD, United States of America; 4 JHU/MWH Alliance, 615 N. Wolfe St., Johns Hopkins University, Baltimore, MD; 5 Statistics Group, Northeast Area Office, Agricultural Research Service, United States Department of Agriculture, Beltsville, MD, United States of America; ICAR- Indian Agricultural Research Institute, INDIA

## Abstract

Organic materials in fresh-cut produce wash water deplete free chlorine that is required to prevent pathogen survival and cross-contamination. This research evaluated water quality parameters frequently used to describe organic load for their fitness to predict chlorine demand (CLD) and chemical oxygen demand (COD), which are major needs identified by the industry-led produce food safety taskforce. Batches of romaine lettuce, iceberg lettuce, or carrot of different cut sizes and shapes were washed in 40 liters of water. Physicochemical properties of wash water including CLD, COD, total organic carbon (TOC), total suspended solids (TSS), total dissolved solids (TDS), turbidity, total sugar content, and pH, were monitored. Results indicate that pH is primarily commodity dependent, while organic load is additionally impacted by cutting and washing conditions. Significant linear increases in COD, TOC, CLD, TDS, and turbidity resulted from increasing product-to-water ratio, and decreasing cut size. Physicochemical parameters, excluding pH, showed significant positive correlation across different cut sizes within a commodity. High correlations were obtained between CLD and COD and between COD and TOC for pooled products. The convenient measurement of TDS, along with its strong correlation with COD and CLD, suggests the potential of TDS for predicting organic load and chlorine reactivity. Finally, the potential application and limitation of the proposed models in practical produce processing procedures are discussed extensively.

## Introduction

Fresh-cut produce wash operations are prone to pathogen cross-contamination when the concentration of sanitizers fall below operation-specific critical limits [1, 2]. Among all sanitizers available, chlorine is most widely used by the fresh-cut produce industry because of its high efficacy against diverse pathogens, and low application cost. However, free chlorine (FC) concentration in wash water decreases rapidly in the presence of organic materials. Thus, fresh-cut produce processors often monitor organic load in wash water as part of their food safety programs. Presently, numerous methods are used, including chemical oxygen demand (COD), total organic carbon (TOC), total dissolved solids (TDS), total suspended solids (TSS), turbidity, and total solid content [3]. While several studies have compared methods for monitoring organic matter in the drinking water industry [4], studies on the suitability of methods commonly used to monitor organic load in produce wash water are scarce. Most importantly, the interrelationships of these physicochemical measurements, and the impacts of commodity type, cut shape and size, and fresh-cut produce wash operating conditions are unknown. In a call for research proposals initiated by the Center for Produce Safety, the produce industry organization asked for reliable tools to monitor organic load and improve operator control over wash water sanitation [5]. In the guidelines to validate control of cross-contamination during washing of fresh-cut leafy vegetables, method standardization and evaluation of organic load in wash water were identified as key data gaps [2]. This research was designed to address these critical research needs.

Among the commonly used water quality parameters, COD is often used by the industry and researchers to monitor water quality changes during fresh-cut washing processes. COD directly measures the oxygen required to oxidize soluble and particulate matters in water. Previous studies have shown that COD increases with increased product loading rate in the same tank of water [6, 7]. The measurement of COD is time-consuming (typically two hours), and currently there is no on-line monitoring method available.

TOC measures the amount of carbon contained in the organic compounds, and is often used as a non-specific indicator of water quality in the drinking water industry [8, 9]. While on-line monitoring of TOC is available, the instruments are costly, and the applications are often limited by their range (e.g., below 50 mg/L). Total soluble solids (°Brix) measures the total amount of sugars contained in water or a solution. Brix can be determined conveniently with a portable refractometer, but the applicability of this parameter to estimate FC consumption needs evaluation, due to the low reactivity of sugars with chlorine [10].

Total dissolved solids (TDS) measures the conductivity arising from soluble substances. It is not a direct measurement of organic load, since sugars as the major organic compounds in wash water exhibit negligible conductivity [11]. However, previous studies have found parallel changes in this parameter with COD [12, 13]. TDS can be measured via simple and inexpensive procedures, and it has been employed as an indicator for chlorine dosing, since the dissolved matter is the main contributor to chlorine consumption in drinking water [14]. Similarly, total suspended solids (TSS) measures the amounts of suspended inorganic (e.g., soil particles) and organic (e.g., produce debris) solids per volume of water, both of which increase along with the total amount of produce washed [15]. Turbidity measures the intensity of light scattered by fine particles in the samples. The measurement is rapid (seconds to minutes), inexpensive, and easy to obtain. However, since both organic and soil particles are capable of scattering light, the suitability and accuracy of turbidity for organic monitoring during fresh produce washing is unknown.

CLD is the amount of FC that a sample depletes, and it indicates directly how much sodium hypochlorite is needed to achieve the desired FC level in a defined period. One limitation of direct CLD measurement lies in its time-intensive procedures. Thus, finding a faster method that correlates well with CLD is important.

In this study, a comprehensive investigation was performed to identify possible water quality parameters as suitable predictors for COD and CLD during fresh produce washing. As will be emphasized in Discussion, the study is relevant to a system with low degrees of chlorination, such an immersion-free processing line or a pre-washing system. The dynamics of the abovementioned parameters will be significantly different in an extensively chlorinated system (e.g., a flume), which will be the subject of a separate study. The main objectives of this study are to 1) evaluate the physicochemical properties of produce wash water as impacted by typical fresh-cut processing operation variations, namely commodity type, cut size, and product loading ratio; 2) determine the interrelationship of major physicochemical parameters of fresh-cut wash water, and the potential to predict COD and CLD using more efficient measurement approaches.

## Materials and methods

### Materials

Romaine lettuce (*Latuca*. *sativa var*. *longifolia*.), iceberg lettuce (*Latuca*. *sativa var*. *capitata*), and carrot (*Daucus carota subsp*. *sativus*) were purchased from a produce wholesale market (Jessup, MD, USA). The produce was transported (within 30 min) to the Food Quality Laboratory in Beltsville, MD, USA, and used within 24 h of storage at 5°C. All chemical reagents used in this study were of analytical grade.

### Preparation of wash water

Produce was cut using an industrial vegetable slicer **(**Nichimo Seven Chefs ECD-302, Tokyo, Japan). Fresh carrots were stick-cut (3 mm x 3 mm x 26 mm) or sliced (3 mm thickness). Fresh romaine and iceberg lettuce heads were shredded (3 mm width) or chopped (26 mm x 26 mm squares) after coring. For each type of produce, one kilogram of the cut product was contained in a nylon mesh bag and dipped in distilled water (20 L for lettuces, or 40 L for carrot) with gentle manual agitation for 1 min. After removing the product from the water and draining into the wash basin, a new batch (1 kg) of product was washed in the same water following the same procedure. Additional fresh water (typically around 50 mL, based on the observed water loss) was supplemented after each wash cycle to ensure consistent water volume. This process was repeated 10 times for carrot and 15 times for lettuce. Water samples were collected before and after each wash, and tested for water quality parameters following the procedures listed in the section below.

### Characterization of wash water

Wash water was characterized by standard EPA methods. The pH was determined with a digital pH meter (Oakton Instruments, Vernon Hills, IL, USA). Turbidity was determined with a turbidity meter (Orion AQ4500, Thermo Scientific, Singapore). TDS was determined with a TDS meter (135A; Thermo Orion, Germany). TSS was determined using EPA method 160.2 [16]. Total sugar content (in degrees of Brix) was measured with a refractometer (PR-101, Spectrum Technologies, Plainfield, IL, USA). COD was determined by mercury-free reactor digestion method [17]. TOC was measured by persulfate-ultraviolet oxidation method (Method 5310) using a Sievers 900 series TOC analyzer (GE Analytical Instrument, Boulder, CO, USA). CLD was determined by Hach method 10223 [18]. In brief, twenty microliters of commercial sodium hypochlorite solution (100–150 g/L, Sigma Aldrich, St Louis, MO, USA) was diluted in 1 mL of the tested samples. After 90 min of mixing, the residual FC level was determined using the DPD colorimetric method [19]. CLD was calculated as the reduction in FC level after incubation.

### Experimental design and statistical analyses

All treatments and measurements were conducted in triplicate with a completely randomized design. Pearson correlation analysis was performed to establish the correlation among various water quality parameters, using Sigma Plot version 12.0 (Systat Inc., Chicago, IL, USA). To identify possible parameters for predicting COD and CLD, quadratic analysis of covariance (ANCOVA) models were fit; first allowing unique parameters for each of the 6 products, and subsequently combining data from both preparation methods for a given produce type when contrast test indicated parameters were statistically similar for the two preparation methods. ANCOVA models were fit using SAS version 9.4 PROC MIXED (SAS Institute, Inc., Cary, NC, USA) [20].

## Results and discussion

### Dynamic changes in wash water quality as impacted by produce cutting and washing operating conditions

**Fig 1:**
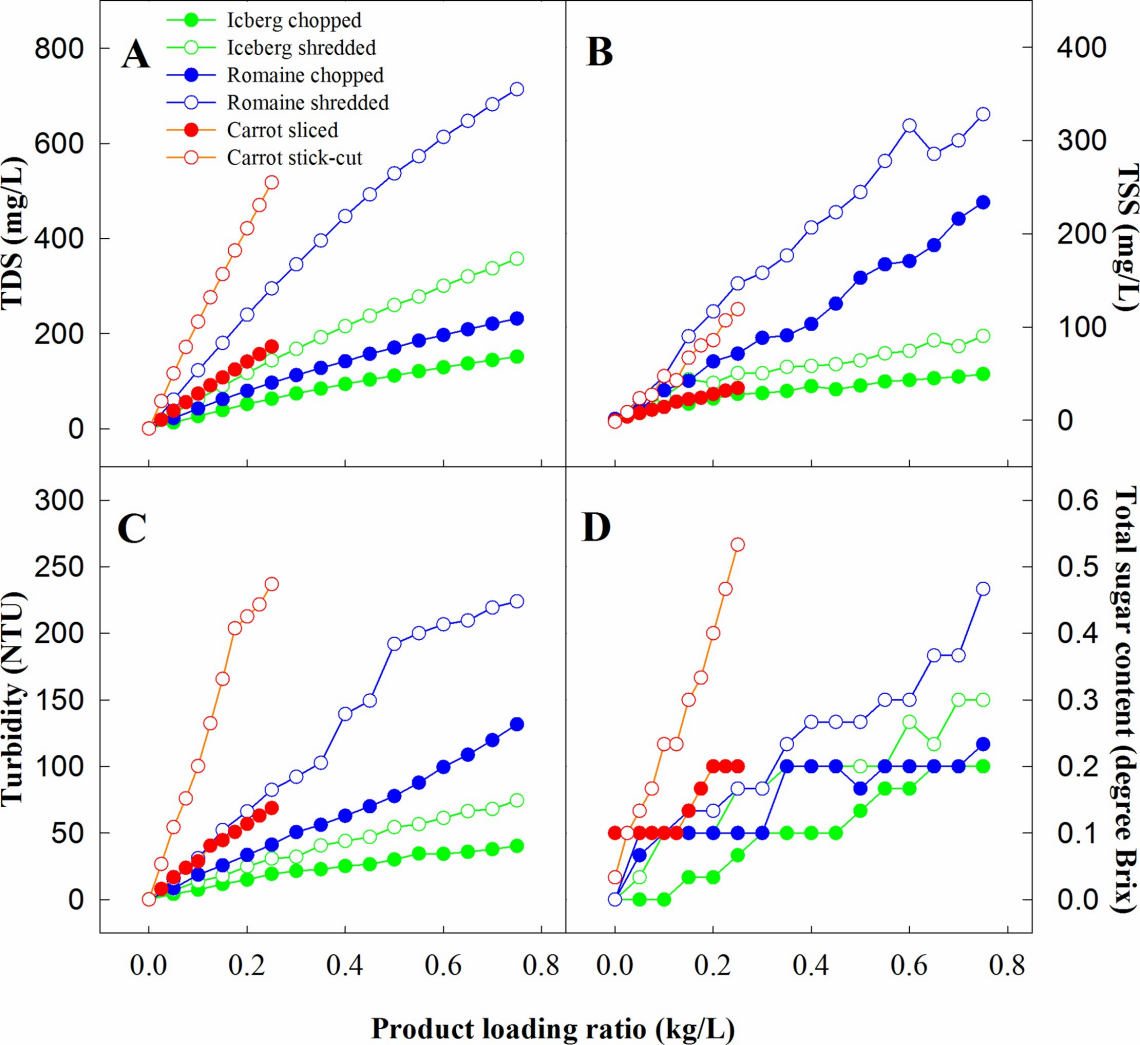
Change, relative to product loading ratio in total dissolved solids (TDS, A), total suspended solids (TSS, B), turbidity (C), and total sugar content (D) in the wash water with fresh product (1 kg per batch) repeatedly washed in the same tank of water.

**Fig 2:**
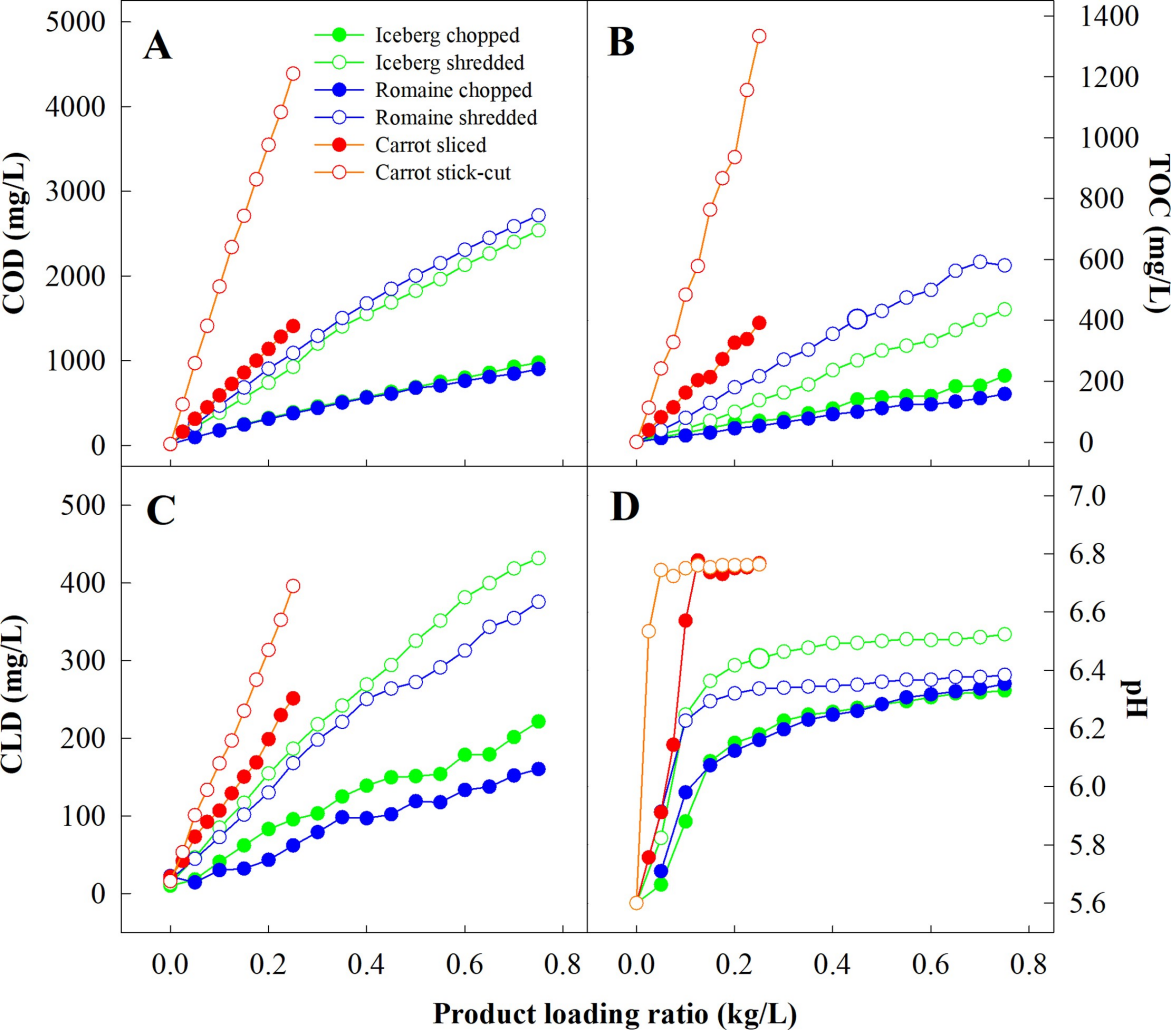
Change, relative to product loading ratio in chemical oxygen demand (COD, A), total organic carbon (TOC, B), chlorine demand (C) and pH (D) of the wash water for different produce types and cut sizes and shapes.

Commodity type, produce loading rate, and cut size and shape significantly affected the dynamic changes of the physicochemical properties of wash water (Figs 1A–1D and 2A–2C). Increasing the amount of product washed in the same tank of water resulted in a nearly linear increase in the values of COD, TOC, TSS, TDS, Brix, and CLD. This observation suggests a continuous accumulation of organic materials in wash water, leading to an increasing capacity for free chlorine depletion. Previous studies from Luo [21] and Weng et al. [22] showed similar findings related to COD, TDS, and turbidity on a limited scope. The pH values (Fig 2D) increased significantly with the first batch of product, followed by gradual increase towards stabilization as more products were washed.

The cut size and shape significantly impacted the rate of water quality decline. For the same commodity, washing shredded product resulted in two to three folds more rapid water quality deterioration than washing the sliced product. The more cut surfaces per unit weight of the shredded products may have contributed to the more rapid release of tissue exudates and thus steeper decline in water quality.

Commodity type also played a significant role in the changes in water quality [12, 21]. Most notably, increasing the loading rate of both shredded and sliced carrots led to a much more rapid increase in brix, COD, TOC, and CLD than that in cut romaine and iceberg lettuce, regardless of cut size and shape (comparing Figs 1D and 2A–2C). Commodity-specific factors such as chemical composition, solubility of the components, and surface rigidity, may have played significant roles in organic load and chlorine demand in wash water. The rapid changes in physicochemical properties in cut carrot wash water might be attributable to the high solids content in carrot juice. Carrot typically contains 11~13% carbohydrates (by fresh weight), including a significant amount (1.67–3.35%) of reducing sugars [23]. Those compounds are the major contributors to brix, TOC, and COD. The CLD observed for carrot wash water is most likely due to significant levels of other highly reactive compounds including phenolics, ascorbic acid, anthocyanins and carotenoids. On the other hand, the slow increase in TSS in carrot wash water was probably ascribed to carrot’s hard texture and higher density, which might have prevented the products from breaking down during washing and remaining in the water after the products were removed.

Comparing the two lettuce types, shredded or chopped romaine lettuce produced higher TDS, TSS, turbidity, brix, and TOC than shredded or chopped iceberg lettuce (Figs 1A–1D and 2B). However, the trend was reversed for CLD where both shredded and chopped romaine had at most 20% lower CLD than iceberg lettuce at the same cut size. There was no difference in COD between shredded and sliced romaine over that of iceberg lettuce. Differences in head morphology result in romaine and iceberg lettuce slices or shreds having slightly different cut size and shape even when produced using the same cutter setting. However, the significantly higher TDS, TSS, turbidity, and brix and lower CLD in romaine than in iceberg lettuce suggest substantially different chemical composition and texture in these two lettuce products. The slightly higher solids content in romaine (5%) than that in iceberg (4%) may have contributed to the higher TDS [23], while the soft texture of romaine lettuce leaves resulted in the formation of more leaf debris and thus higher TSS. Turbidity is a rather complex parameter that can be influenced by a multitude of factors, including solid content, abundance and solubility of macromolecules such as proteins, interaction between proteins and phenols, ionic strength of the water, and pH relative to the isoelectric point of proteins [24]. Collectively, these factors are responsible for the higher turbidity in romaine lettuce wash water than in iceberg lettuce wash water.

Interestingly, although wash water from romaine had higher TOC than that from iceberg lettuce, the former did exhibited a comparable COD and significantly lower CLD than the latter. TOC measures merely the amount of carbon atoms. The higher TOC in romaine lettuce wash water was possibly attributable to the greater amount of carbohydrate and protein, which are the major sources of carbon atoms [23]. On the other hand, COD is affected by more factors as it reflects the number of all oxidizable atoms, mainly carbon, hydrogen, nitrogen, and sulfur. Therefore, the disparate results in TOC and COD is attributable to the different chemical compositions of the two lettuce varieties. CLD is more complex than TOC and COD, as CLD is determined not only by the number of atoms in the system but also by their arrangement, which dictates their reactivity. For instance, iceberg lettuce contains a significantly higher amount of ascorbic acid (4.2 mg/100 g), a good substrate for FC [10], than does romaine lettuce (2.8 mg/100 g) [25]. In addition, nearly half of the solids content in romaine lettuce is dietary fiber (oligo-or polysaccharides) [23], which contributes significantly to the TOC but is less reactive with FC than monosaccharides due to its lower water solubility. A systematic comparison of potential chlorine-depleting compounds and their quantities in these two lettuce types may provide more insight into the chlorine-produce interactions.

### Correlation among wash water quality parameters

**Fig 3:**
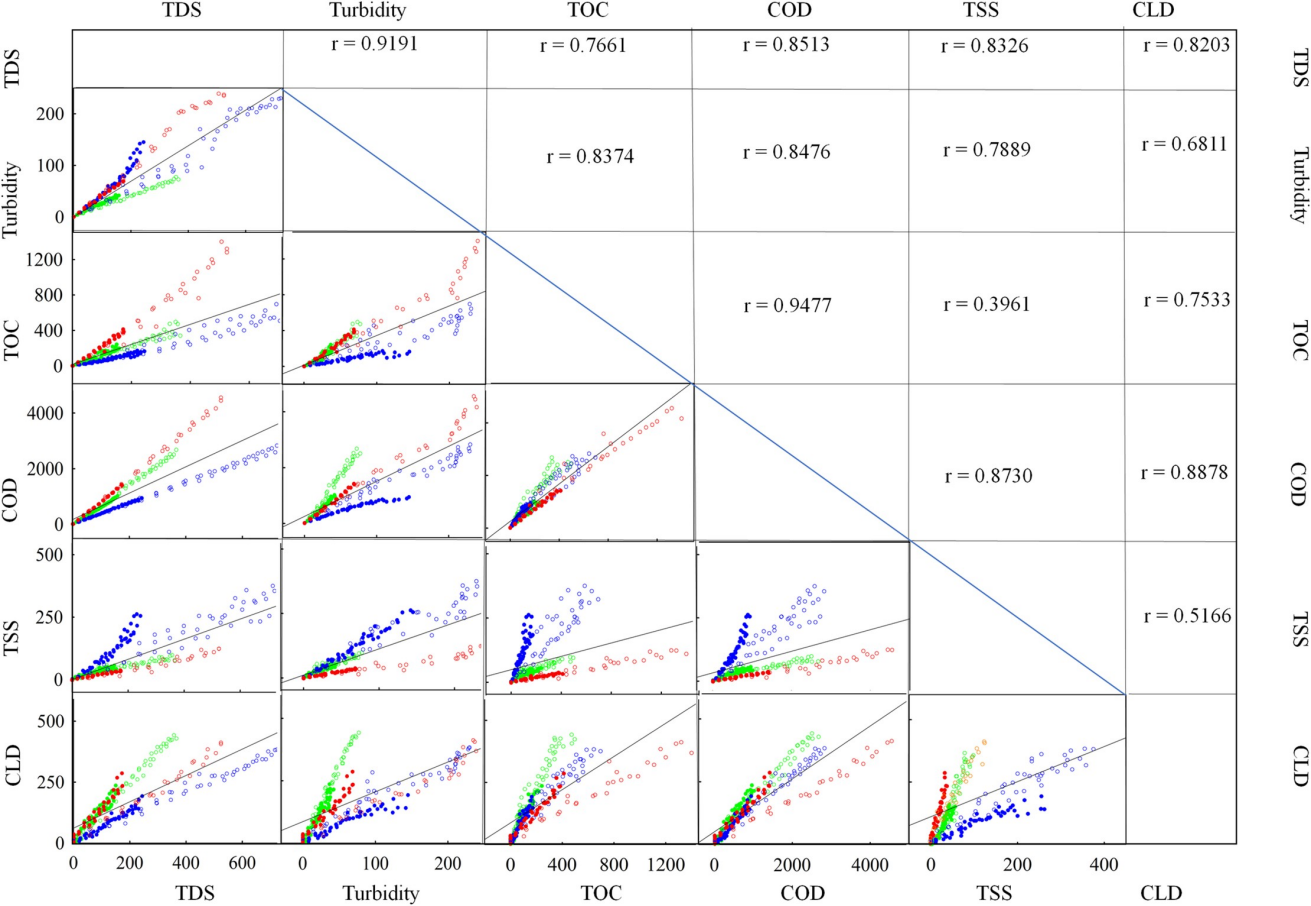
Matrix of Pearson linear regressions and Pearson correlations (r) among each pair of chemical oxygen demand (COD), total organic carbon (TOC), chlorine demand (CLD), turbidity, total dissolved solids (TDS), and total suspended solids (TSS). All product types are pooled in each chart.

Fig 3 presents the correlations among major physicochemical parameters with six products combined (2 cut sizes for each of the 3 types of produce). Since the goal of this study was to find predictors for CLD and COD, we looked at the parameters that had the strongest correlations with CLD and COD. COD (r = 0.8878) and TDS (r = 0.8203) showed the strongest correlations with CLD. On the other hand, CLD showed the weakest correlation with TSS (r = 0.5166). COD was strongly correlated with all the water quality parameters other than pH with the order of greatest to least correlated parameters being TOC (r = 0.94770), CLD (r = 0.8878), TSS (r = 0.8730), TDS (r = 0.8513) and turbidity (r = 0.8476). Although TOC was highly correlated to COD, its correlation to CLD was much poorer (r = 0.7533). Among other parameters, TDS was highly correlated with turbidity (r = 0.9191), and TSS (r = 0.8326), but least correlated with TOC (r = 0.7661) The least correlated parameters were TSS and TOC (r = 0.3961). These results suggest that COD is the best predictor of CLD, followed by TDS, while TOC is the best predictor of COD, with all the other parameters being only slightly less apt.

**Fig 4:**
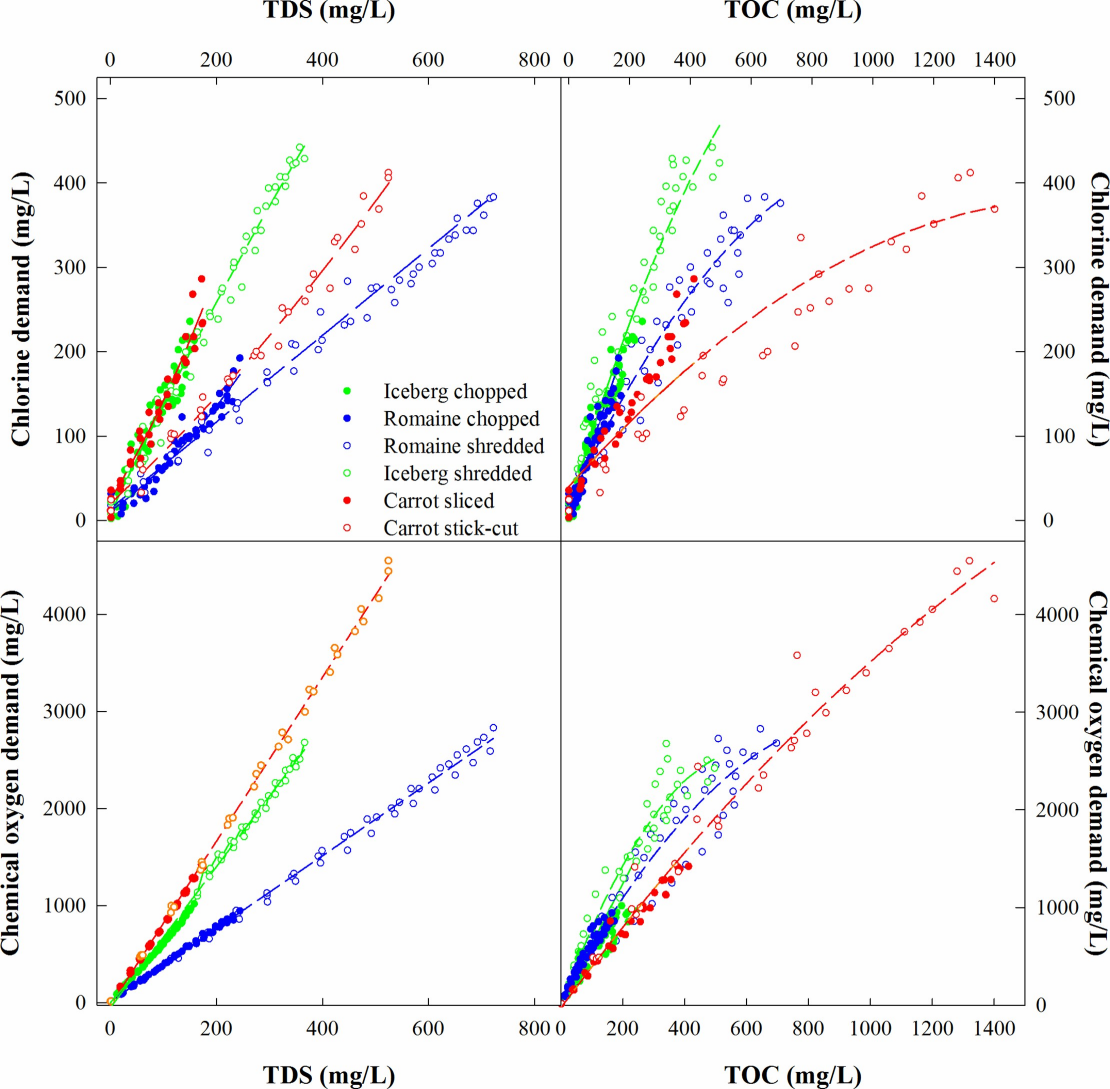
Comparison between linear and polynomial regression models for selected water quality parameters.

Based on the above correlations and visual assessment in Fig 3, quadratic regression models were specified within a framework of four ANCOVA models to predict each of CLD and COD using each of TDS or TOC. Each ANCOVA model was initially constructed by specifying a distinct quadratic regression model for each of the 6 unique products (S1 Table). When the regression models’ parameters were statistically indistinguishable between the two preparation methods for a specific produce type, one regression model was fit to the combined data observed for both preparation methods (Table 1). R^2^ values, all greater than 0.93, suggest TDS and TOC can be used to accurately predict CLD and COD. The measured and modeled CLD and COD values are illustrated in Fig 4.

**Table 1:** Quadratic predictive models for CLD and COD regressed onto TDS or TOC.

Product type	ANCOVA Regression Models
R^2^ = 0.982	***CLD***
Iceberg [chopped & shredded]	***=*** - 0.000370∙***TDS***^***2***^ ***+*** 1.327∙***TDS +*** 8
Romaine chopped	***=*** 0.000909∙ ***TDS***^***2***^ ***+*** 0.441∙***TDS +*** 11
Romaine shredded	= 0.514∙TDS + 14
Carrot sliced	***=*** 0.001715∙ ***TDS***^***2***^ ***+*** 1.001∙***TDS +*** 25
Carrot stick-cut	***=*** 0.000268∙ ***TDS***^***2***^ + 0.583∙***TDS*** + 20
	
R^2^ = 0.933	***CLD***
Iceberg [chopped & shredded]	***=*** -0.00061∙ ***TOC***^***2***^ ***+*** 1.218∙***TOC +*** 14
Romaine chopped	***=*** 1.07 ∙***TOC +*** 9
Romaine shredded	***=*** -0.00035∙***TOC***^***2***^ ***+*** 0.762∙***TOC +*** 20
Carrot [sliced & stick-cut]	***=*** -0.00012∙***TOC***^***2***^ ***+*** 0.405∙***TOC +*** 40
	
R^2^ = 0.998	***COD***
Iceberg chopped	***=*** 6.247∙***TDS***
Iceberg shredded	***=*** 7.255∙***TDS—***50
Romaine [chopped & shredded]	***=*** 3.748∙***TDS +*** 14
Carrot [sliced & stick-cut]	***=*** 8.448∙***TDS—***26
	
R^2^ = 0.969	***COD***
Iceberg chopped	***=*** 6.14∙***TOC +*** 18
Iceberg shredded	***=*** -0.0068 ∙***TOC***^***2***^ ***+*** 8.42∙***TOC +*** 17
Romaine [chopped & shredded]	***=*** -0.00284∙***TOC***^***2***^ ***+*** 5.77∙***TOC +*** 58
Carrot [sliced & stick-cut]	***=*** -0.00071∙***TOC***^***2***^ ***+*** 4.25∙***TOC—***23

The R^2^ values indicate the proportion of the total observed CLD or COD variability described by the collection of quadratic regression models composing each of the 4 ANCOVA models. For a given type of produce, a combined model is provided (e.g., combining chopped and shredded; or combining sliced and stick-cut) when the contrast test indicates that individual models for the two separate preparation methods were statistically indistinguishable.

### Additional discussion

#### Chemical composition dictates the correlation among water quality parameters

In the fresh produce processing industry, the term “organics” or “organic load” has been commonly used to represent the potential consumption of FC during washing, and the level of organic load is typically characterized by COD. For the same type of produce, all its constituent compounds accumulate simultaneously as the product load increases, regardless of their contribution to whichever parameter. This accounts for the parallel increase in all measured parameters (except for pH), as well as the strong, positive correlation thereof. However, when different types of produce were compared, the type that generates higher COD or TOC during washing does not necessarily cause greater CLD, which leads to different conversion factors and prediction models among diverse types of produce.

It has been well established that the chlorination kinetics of various compounds (e.g., sugars, amino acids, proteins, and phenols) depends on its abundance and reaction rate constant [26]. Sugars are the most abundant type of compound in most types of produce [23]. They are probably the major contributor to COD and TOC in the wash water, but their chlorine demand needs to be evaluated because of their low reaction rate [10]. On the other hand, compounds that have limited contribution to COD or TOC may result in a considerable CLD. For instance, proteins and peptides are found in low abundance in the wash water [22]. However, the reactive groups (thiol, amine, carboxylic, etc.) on their side chains react with chlorine at a rate several orders of magnitude higher than sugars. Therefore, their contribution to CLD may still be substantial [26]. In addition, the reactivity of proteins and peptides is highly affected by the amino acid composition, conformation, and molecular weight [27]. Therefore, two types of produce generating the same amount of proteins or peptides in wash water are not guaranteed to incur the same level of CLD. Finally, as biomacromolecules, proteins are prone to forming aggregates under favorable conditions, such as acidic pH [28] and the presence of polyphenols. This phenomenon probably contributes to the turbidity of the wash water samples.

Organic acids contribute substantially to TDS due to their ionization, and they react moderately with FC. The reaction rate varies substantially by the type acid [10]. Phenolic compounds are the third most abundant type of compounds in many types of produce, whose content can reach as much as 0.4 mg/g fresh weight in red and orange carrots [29]. However, their limited solubility should be taken into consideration when assessing their chlorine demand during washing. For instance, most phenolic acids in lettuce were found in the conjugated form, which is less reactive than their free soluble counterparts [30]. Finally, debris that is rich in fiber and other insoluble matter may be the major source of TSS, but its chlorine demand is expected to be rather limited.

In summary, different water quality parameters reflect the accumulation of various compounds. A more comprehensive study on the chemical composition and chlorine demand of fresh produce wash water will be covered in an upcoming research article. Nevertheless, due to the synchronic increase in those parameters during washing, one parameter could potentially serve as a predictive indicator of another, as was found in this study.

#### Limitation of the proposed predictive model

A major limitation of the predictive model shown in Table 1 lies in different dynamics of various water quality parameters during chlorination. In this study, all parameters were measured without chlorination, and those parameters were basically a function of the loading ratio. Whereas, in conventional produce washing systems such as flumes, the wash water is constantly chlorinated to overcome the CLD. The degree of chlorination has a substantial effect on the dynamics of all water quality parameters, as discussed below. During chlorination, the CLD of wash water is apparently reduced, and the extent of reduction equals approximately the amount of added FC. However, TOC is not expected to change in the same pattern upon chlorination. Only a small percentage of carbon atoms is released as volatile chlorination byproduct; therefore, the total carbon content of the wash water remains largely unchanged. TDS increases in chlorinated wash water because of the partial ionization of hypochlorous acid and certain chlorination byproducts (e.g., haloacetic acids) [31], in contrast to the decreasing CLD. Finally, the suitability of turbidity for predicting CLD in chlorinated wash water is questionable as turbidity is influenced not only by the content but also by the structure of proteins/peptides in the system, the latter of which may be affected by chemical reactions occurring on their side chains. All those facts suggest the divergence of CLD and other water quality parameters as FC is introduced at significant levels, posing a challenge to the prediction methods presented in this study.

COD, on the other hand, may still be predicted accurately by TOC. Previous studies revealed minimal change in COD in fresh produce wash water upon reconditioning with FC [32]. This is probably attributed to the fact COD in fresh produce wash water originates mainly from carbohydrates [11], which remain mostly intact in the presence of FC. The fact that both COD and TOC remain relatively stable during chlorination allows the prediction of one with another.

Based on the above consideration, it is recommendable to predict CLD using the developed models only under low chlorination levels (i.e., the remaining CLD in the system is close to the theoretical maximum determined without added FC). For instance, the models may be applied on a pre-washing system equipped with sprayers. The relatively low FC in the sprayed water and short contact time limit the influence of chlorination, making it possible to predict the CLD of the water collected from the pre-washer. The predicted value could further be converted by a proper volumetric factor to estimate the amount of FC required in the flume. Likewise, water sample may be collected from an immersion-free processing system [33]. The CLD may be predicted by parameters such as TOC and TDS, providing feedback for FC level maintenance.

There are alternative parameters that can potentially predict CLD in more extensively chlorinated wash water. For instance, measurement of UV absorbance at two distinct wavelengths provides information of both total and achieved CLD in a water sample, thus providing valuable information on the residual CLD needed to be overcome during washing [34]. Following the same rationale, infrared spectroscopy also holds potential of differentiating achieved and total CLD, in that the absorbance at different wavenumbers may provide information on compounds with high (e.g., proteins and peptides) and low (e.g., sugars) reactivities with sugars [35].

## Conclusions

Comprehensive evaluation of fresh-cut produce wash water physicochemical properties as impacted by processing condition, commodity type, and their correlations was performed. Romaine lettuce wash water had higher total suspended solids, total dissolved solids, turbidity and total organic carbon than iceberg lettuce wash water. However, these did not translate into higher chlorine demand or chemical oxygen demand. Strong positive correlations were identified between CLD and TDS, CLD and TOC, COD and TDS, and COD and TOC. The correlative patterns were identified as produce type dependent for each pair of the above parameters, per quadratic ANCOVA. Therefore, individual quadratic models were established for each produce type to predict CLD and COD from TDS and TOC. These parameters provided a more convenient yet still accurate (R^2^ values greater than 0.95) means of predicting CLD for each type of produce. This is the first systematic study on the dynamic assessment of fresh produce wash water under simulated water reuse conditions. The results are useful for the fresh-cut produce industry for designing better systems and testing parameters to monitor wash water quality and predict chlorine demand for different produce commodities. The presented method is anticipated to be most reliable in immersion-free processing lines and pre-washing systems. Consideration should be made when this method is applied in fully chlorinated systems, such as flumes, as the dynamic change in water quality parameters is altered significantly at higher degrees of chlorination.

## Supporting information

S1 Table Individual regression models for predicting chlorine demand (CLD) and chemical oxygen demand (COD) with total dissolved solids (TDS) and total organic carbon (TOC). (DOCX)
