# Supplementary material for: Dynamic changes in the physicochemical properties of fresh-cut produce wash water as impacted by commodity type and processing conditions
Source: PLoS One. 2019 Sep 26;14(9):e0222174. doi: 10.1371/journal.pone.0222174 (PMC6762053; doi:10.1371/journal.pone.0222174)
Supplement: S1 Table — (DOCX) [file pone.0222174.s001.docx]

**S1 Table** Individual regression models for predicting chlorine demand (CLD) and chemical oxygen demand (COD) with total dissolved solids (TDS) and total organic carbon (TOC). Four sets of models were established to predict CLD with TDS, CLD with TOC, COD with TDS, and COD with TOC, respectively. Within each set, six models were presented for all six product types. The R^2^ value for each set describes the overall accuracy of the included six models.

| **Produce type** | **Cut shape** | **Model** |
| --- | --- | --- |
| *CLD = f (TDS), R^2^=0.983* | | |
| Romaine | Chopped | CLD = 11 + 0.441 x TDS + 0.000901 x TDS^2^ |
|  | Shredded | CLD = 14 + 0.514 x TDS |
| Iceberg | Chopped | CLD = 6 + 1.396 x TDS |
|  | Shredded | CLD = 8 + 1.262 x TDS |
| Carrot | Sliced | CLD = 25 + 1.001 x TDS |
|  | Stick-cut | CLD = 20 + 0.582 x TDS + 0.000268 x TDS^2^ |
| *CLD = f(TOC), R^2^=0.961* | | |
| Romaine | Chopped | CLD = 9 + 1.067 x TOC |
|  | Shredded | CLD = 20 + 0.762 x TOC – 0.00035 x TOC^2^ |
| Iceberg | Chopped | CLD = 8 + 1.311 x TOC – 0.00175 x TOC^2^ |
|  | Shredded | CLD = 22 + 1.376 x TOC – 0.00106 xTOC^2^ |
| Carrot | Sliced | CLD = 26 + 0.482 x TOC |
|  | Stick-cut | CLD = 20 + 0.336 x TOC |
| *COD = f (TDS), R^2^=0.998* | | |
| Romaine | Chopped | COD = 12 + 3.804 x TDS |
|  | Shredded | COD = 3.771 x TDS |
| Iceberg | Chopped | COD = 6.247 x TDS |
|  | Shredded | COD = -50 + 7.255 x TDS |
| Carrot | Sliced | COD = -2 + 8.084 x TDS |
|  | Stick-cut | COD = -13 + 8.432 x TDS |
| *COD= f(TOC), R^2^=0.970* | | |
| Romaine | Chopped | COD = 8 + 7.84 x TOC |
|  | Shredded | COD = 48 + 5.79 x TOC – 0.00284 x TOC^2^ |
| Iceberg | Chopped | COD = 18 + 6.14 x TOC |
|  | Shredded | COD = 17 + 8.42 x TOC – 0.00681 x TOC^2^ |
| Carrot | Sliced | COD = 13 + 3.88 x TOC |
|  | Stick-cut | COD = 32 + 4.36 x TOC – 0.00086 x TOC^2^ |

Note: Quadratic terms were omitted from the model when the associated coefficient was statistically indistinguishable from zero.
